# Mosquito-borne arbovirus surveillance at selected sites in diverse ecological zones of Kenya; 2007 – 2012

**DOI:** 10.1186/1743-422X-10-140

**Published:** 2013-05-10

**Authors:** Caroline Ochieng, Joel Lutomiah, Albina Makio, Hellen Koka, Edith Chepkorir, Santos Yalwala, James Mutisya, Lillian Musila, Samoel Khamadi, Jason Richardson, Joshua Bast, David Schnabel, Eyako Wurapa, Rosemary Sang

**Affiliations:** 1 Division of Emerging Infectious Disease, United States Army Medical Research Unit, Kenya, P. O. Box 606, Village Market, Nairobi, Kenya; 2 Centre for Virus Research, Kenya Medical Research Institute, P. O. Box 54628, Nairobi, Kenya; 3 International Centre for Insect Physiology and Ecology, P. O. Box 30772–00100, Nairobi, Kenya; 4 Walter Reed Army Institute of Research, Silver Spring, USA

**Keywords:** Arbovirus, Surveillance, Mosquitoes

## Abstract

**Background:**

Increased frequency of arbovirus outbreaks in East Africa necessitated the determination of distribution of risk by entomologic arbovirus surveillance. A systematic vector surveillance programme spanning 5 years and covering 11 sites representing seven of the eight provinces in Kenya and located in diverse ecological zones was carried out.

**Methods:**

Mosquitoes were sampled bi-annually during the wet seasons and screened for arboviruses. Mosquitoes were identified to species, pooled by species, collection date and site and screened for arboviruses by isolation in cell culture and/or RT-PCR screening and sequencing.

**Results:**

Over 450,000 mosquitoes in 15,890 pools were screened with 83 viruses being detected/isolated that include members of the alphavirus, flavivirus and orthobunyavirus genera many of which are known to be of significant public health importance in the East African region. These include West Nile, Ndumu, Sindbis, Bunyamwera, Pongola and Usutu viruses detected from diverse sites. Ngari virus, which was associated with hemorrhagic fever in northern Kenya in 1997/98 was isolated from a pool of *Anopheles funestus* sampled from Tana-delta and from *Aedes mcintoshi* from Garissa. Insect only flaviviruses previously undescribed in Kenya were also isolated in the coastal site of Rabai. A flavivirus most closely related to the Chaoyang virus, a new virus recently identified in China and two isolates closely related to Quang Binh virus previously unreported in Kenya were also detected.

**Conclusion:**

Active transmission of arboviruses of public health significance continues in various parts of the country with possible undetermined human impact. Arbovirus activity was highest in the pastoralist dominated semi-arid to arid zones sites of the country where 49% of the viruses were isolated suggesting a role of animals as amplifiers and indicating the need for improved arbovirus disease diagnosis among pastoral communities.

## Background

Arboviruses constitute a heterogeneous group of mostly zoonotic viruses transmitted by hematophagous arthropods, including mosquitoes, ticks and sand flies [1]. Most of these viruses are maintained in zoonotic cycles and humans are usually incidental dead-end host with an insignificant role in maintaining the cycle of the virus [2]. They cause clinical syndromes of varying severity in humans and animals, ranging from self-limiting febrile illnesses to life-threatening encephalitis and/or hemorrhagic fever in humans and overt to severe/fatal disease in animals [2]. They replicate in the arthropods that become infected following a blood meal on viremic vertebrate hosts [2]. Virus isolation from human clinical specimens is usually difficult since the virus is only transiently present in blood during the viremic phase.

Arbovirus infection can be most effectively controlled by use of vaccines. However, this is limited by lack of registered vaccines for the majority of circulating arboviruses in the region except the Yellow Fever vaccine and Rift Valley Fever vaccine for livestock use only. Early detection of virus activity or detection of increased virus activity in the vector populations can be a key indicator or early warning for appropriate action to reduce outbreaks. Surveillance programs designed to monitor virus activity in vectors also provides a system for mapping disease distribution and information needed not only to assess risk but also to identify vector species for targeted control [3]. To assess the geographic distribution of these viruses, arthropods are usually a viable alternative surveillance target because the viral infection in the vector is sustained for life. During periods of increased reservoir, vector and humans/domestic animals contact precipitated by suitable environmental, socio-economic conditions/activity there is increased prevalence of infection in vector mosquitoes [4] and subsequent human/animal infections and/or amplification resulting in reports of outbreaks or disease incidence in humans and/or animals [3]. As part of continuing entomologic arbovirus surveillance conducted in Kenya by the United States Army Medical Research Unit-Department of Emerging Infectious Diseases (USAMRU-DEID) and the Kenya Medical Research Institute’ Centre for Virus Research. Mosquitoes were collected and analyzed for arboviruses in eleven selected areas in Kenya as a means of assessing arbovirus distribution, the risk of human exposure to arbovirus disease and the potential for disease outbreaks and incriminating associated vectors for targeted control. The surveillance also provided an opportunity to identify new arboviruses or variants of existing arboviruses. The overarching objective of the study was to determine the distribution of arboviruses circulating in selected parts of Kenya and identify the associated mosquito vectors. This paper reports the arboviruses that were isolated and their associated vectors during the period 2007 to 2012.

## Results

### Mosquito collection and pooling

A total of 450,680 mosquitoes were collected mostly by CDC Light trapping. 15,890 mosquito pools each containing up to 25 mosquitoes, were tested by cell culture screening and 158 pools of *Aedes aegypti* screened by RT-PCR. The highest number of mosquitoes sampled, pooled and tested were from Garissa where the predominant mosquito species (3,454 pools) were flood water *Aedes* species (specifically, *Aedes mcintoshi*, *Aedes ochraceous* and *Aedes tricholabis*) followed by Marigat where the predominant species (1,737 pools) were *Mansonia africana*, *Mansonia uniformis* and *Culex pipiens*. Naivasha (1,111 pools) and Kisumu (514 pools) at the basins of Lakes Naivasha and Victoria respectively were in third place with a predominance of *Cx. pipiens*, *Mansonia* species and *Culex zombaensis*.

### Virus isolation and identification

Overall 84 virus isolates giving reproducible CPE were obtained and characterized by RT-PCR and partial sequencing. They included Alphaviruses (53) among them Sindbis (SINV), Ndumu (NDUV), Babanki (BBKV) and uncharacterized alphaviruses; Flaviviruses (23) including West Nile (WNV), Usutu (USUV) and Insect Flaviviruses; Orthobunyaviruses (8) including Bunyamwera, Pongola and Ngari viruses.

The highest number of virus isolates was detected in mosquitoes sampled in Garissa and mostly from the flood water *Aedes* species; *Ae. mcintoshi* (18), *Ae. ochraceous* (8) and *Ae. tricholabis* (6). Viruses isolated were mostly Alphaviruses including Ndumu virus (NDUV) (94% identity) from *Aedes mcintoshi* (2), *Ae. ochraceous* (2) and *Ae. tricholabis* (3), Babanki virus (94% identity) (from *Ae. mcintoshi* (2) and *Ae. ochraceous* (2)) and unidentified Alphaviruses (7) from *Ae. mcintoshi* and *Ae*. *tricholabis* (2). There were also 4 orthobunyaviruses from this area consisting of one Ngari virus (96% identity), one Pongola and two Bunyamwera viruses (99% identity) isolated from *Ae. mcintoshi*. In addition, isolates of West Nile virus (97% identity) were obtained from 5 pools of *Culex univittatus*. The second highest number of isolates (11) were obtained from samples collected from Marigat with 6 of these being NDUV (94% identity) isolated from *Culex rubinotus* and the other 5 isolated from other *Culex* and *Mansonia* species (Additional file 1).

Around the lake basins, five virus isolates were obtained from samples collected from Kisumu, 7 from Busia and 4 from Naivasha. They consisted of 7 Ndumu isolates (94% identity) from Busia; Sindbis virus from *Cq. fuscopennata* (1), Sindbis-like viruses (94% identity) from *Culex* species (2) and one isolate of Usutu virus (97% identity) obtained from *Cx. pipiens* from Kisumu. Sindbis-like virus (2) isolates and one Babanki virus (98%) isolate were obtained from samples from Naivasha (Additional file 1). Along the coast, seven virus isolates were obtained from samples from Rabai, 3 were identified as insect flaviviruses, 2 were most closely related to Quan Binh Virus (QBV - 82%) recently isolated in China and one was similar to the cell fusing agent virus (CFA – 90%). One (1) Flavivirus isolate from *Aedes aegypti* collected from Rabai was found to be most closely related to a new flavivirus named Chaoyang virus (CYV) with 77% identity. This virus was recently isolated from mosquitoes sampled in China. Three unidentified flaviviruses from *Ae. aegypti* (2) and *Eretmapodite* quinquevittatus (1) and one unidentified alphavirus from *Ae. aegypti* were also isolated in Rabai.

**Figure 1:**
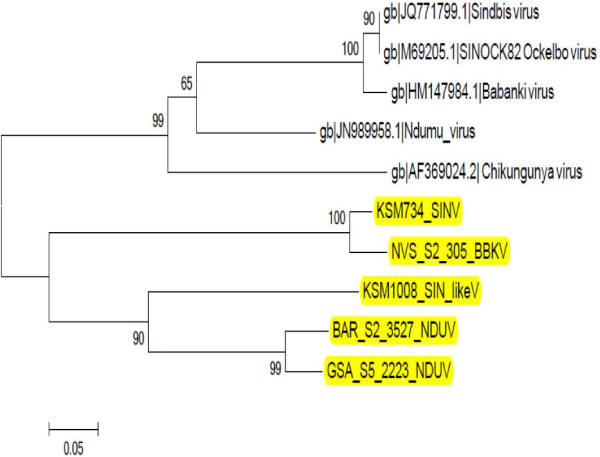
**Neighbour joining phylogenetic tree of nucleotide sequences of selected Alphavirus isolates and reference sequences.** The sequences of the isolates reported in this paper (highlighted in yellow) were compared to other members of the alphavirus genus from Genbank [GenBank: JN989958.1, GenBank: JQ771799.1, GenBank: M69205.1, GenBank: HM147984.1, GenBank: AF369024.2]. The Non structural Protein 4 (NSP4) coding regions of these nucleotide sequences, and those of the selected Alphaviruses in this study were aligned using clustalW and the phylogenetic tree constructed using MEGA v5.05. Numbers on internal branches indicate bootstrap values for 1000 replicates. The results show that the Alphaviruses have grouped into two with one group having the isolates under study (from Kenya) and the other has the reference sequences (from other countries). Within the Kenyan group, the Ndumu virus isolates have clustered separately from the Sindbis and the Sindbis-like viruses. This is due to the diverse nature of Alphaviruses. GSA: Garissa; KSM: Kisumu; NVS: Naivasha; BAR: Baringo. NDUV: Ndumu virus; SINV: Sindbis virus; BBKV: Babanki virus.

**Figure 2:**
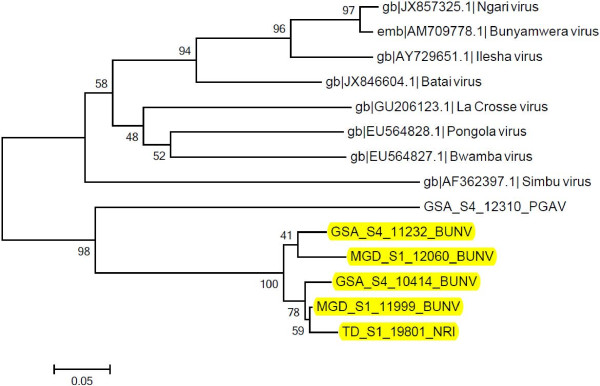
**Neighbour joining phylogenetic tree of nucleotide sequences of selected Orthobunyavirus isolates and reference sequences.** The sequences of the Orthobunyavirus isolates under study (highlighted in yellow) were compared to other Orthobunyaviruses from the database [GenBank: EU564828.1, GenBank: EU564827.1, GenBank: JX857325.1, EMBL: AM709778.1, GenBank: AY729651.1, GenBank: JX846604.1, GenBank: GU206123.1, GenBank: AF362397.1]. The nucleocapsid region of these sequences and those of the selected Orthobunyaviruses under study were aligned using clustalW and the phylogenetic tree constructed using MEGA v5.05. Numbers on internal branches indicate bootstrap values for 1000 replicates. The Orthobunyaviruses from Kenya have clustered together while the reference sequences have also formed a separate clade. Bunyamwera and Ngari viruses have formed a cluster meaning they are closely related while Pongola virus seems to be different from the two viruses. GSA: Garissa; MGD: Magadi; TD: Tana Delta; S: site; PGAV: Pongola virus; BUNV: Bunyamwera virus; NRI: Ngari.

**Figure 3:**
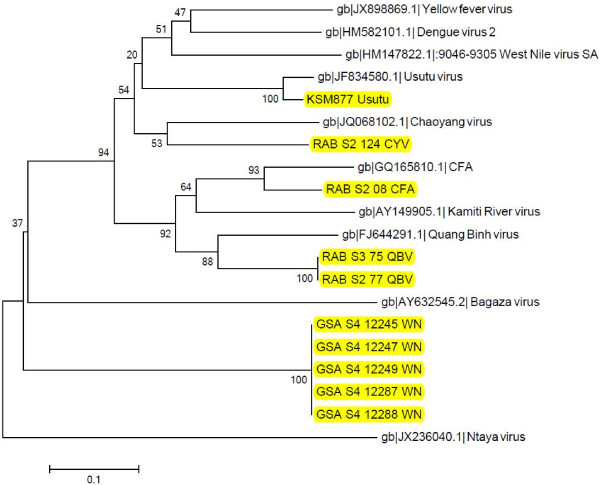
**Neighbour joining phylogenetic tree of nucleotide sequences of selected Flavivirus isolates and reference sequences.** The sequences of the isolates under study (highlighted in yellow) were compared to other Flaviviruses from database [GenBank: HM147822.1, GenBank: JF834580.1, GenBank: GQ165810.1, GenBank: FJ644291.1, GenBank: AY149905.1, GenBank: JQ068102.1, GenBank: HM582101.1, GenBank: JX898869.1, GenBank: AY632545.2, GenBank: JX236040.1]. The NSP5 coding regions of the sequences and of selected Flaviviruses under study were aligned using clustalW and the phylogenetic tree constructed using MEGA v5.05. Numbers on internal branches indicate bootstrap values for 1000 replicates. The Flaviviruses have originated from a common ancestor. The West Nile viruses isolates under study have all clustered together while the Ntaya virus is an outgroup. The insect flaviviruses seems to have recently emerged as compared to other Flaviviruses and they have formed a clade except Chaoyang virus which has fallen to a different clade. Usutu virus is related to West Nile, Dengue and Yellow Fever viruses. GSA: Garissa; KSM: Kisumu; RAB: Rabai; S: site; CYV: Chaoyang virus; QBV: Quang Bihn virus; CFA: Cell fusing agent; WN: West Nile.

Another isolate of Ngari virus was isolated from a pool of *An. funestus* from Tana-delta while isolates of Bunyamwera virus (99% identity) were obtained from 2 pools of *An. funestus* from Magadi. A single isolate of WNV from the *Cx. uinivittatus* was obtained from Turkana. The phylogenetic analyses of the three groups of viruses are available in Figures 1, 2, 3.

## Discussion

The importance of mosquitoes in arbovirus disease transmission and maintenance in East Africa cannot be over-emphasized. Mosquito-borne arbovirus diseases have caused outbreaks afflicting both human and livestock with devastating public health and economic consequences in recent times [2,5] Implementation of mosquito based arbovirus surveillance is therefore vital as part of an early warning systems that could provide information necessary for a rapid response plan against emerging arboviruses or emerging vector/virus associations of public health importance. Surveillance systems also provide opportunities for improvement of our understanding of the ecology and epidemiology of these viruses and the trends in emergence of variants of existing and novel viruses which is key to improving control and preventive measures. Most of the viruses isolated in this study have been associated with human illness. Ndumu virus has been found throughout Africa, and although antibodies to the virus have been identified in humans from several African countries, no human illnesses have been attributed to Ndumu virus infection as such [1,6]. However, Sindbis virus has been isolated from Africa, Australia, Northern Europe, and the Middle East, and symptoms of infection in humans include fever, arthritis, and rash [1]. Babanki, a strain of sindbis virus has been associated with human febrile illness accompanied by rash and arthritis and the virus has been isolated from humans in Cameroon, Madagascar, and the Central African Republic [1,7]. Both Bunyamwera and Pongola viruses are frequently found throughout Africa. Pongola virus has been isolated from a febrile patient in Uganda and specific antibodies have been identified in humans in some African countries [1,8]. Ngari virus has been isolated from two hemorrhagic fever cases (one in Kenya and the other in Somalia) and subsequently, there was additional evidence linking this virus to up to 27% of the hemorrhagic fever cases tested in the 1997/98 RVF outbreak [9]. Usutu virus was first isolated from *Culex naevei* in South Africa in 1959 [10] and was subsequently found in Australia affecting mainly birds [11,12] and subsequently spreading to other European countries. The first severe human infections by Usutu virus were reported in Italy in 2009 causing severe neurological impairment in patients who had had conditions that resulted in immunological impairment [13,14]. The first isolation of Usutu virus from humans was reported from Central Africa Republic from a patient with fever and rash and in Bukina Faso from a patient with fever and jaundice in the 1980s [15]. West Nile virus was first isolated in association of human disease in the West Nile province of Uganda and has since been associated with numerous cases of human disease in Africa, Asia, Europe and the Americas [16]. The findings here suggest the need to include some of the viruses as differentials in the diagnosis of fevers of unknown origin in parts of Kenya.

The sampling in all sites targeted the rainy season when vector abundance is usually expected to be highest resulting in increased host/reservoir and vector contact hence increased virus activity. The sites with the highest mosquito abundance were also sites with the highest number of viruses isolated (Garissa and Marigat). These also happen to be the sites that were the hotspots for Rift Valley Fever outbreak in 1997/98 (Garissa) and 2006/07 (Garissa and Marigat). These two regions are also classified as semi-arid to arid and are inhabited by communities whose economic main stay is pastoralism. Most of the viruses were isolated from the most prevalent mosquito species in Garissa which were the flood water *Aedes* mosquitoes. These species were also associated with Rift Valley Fever virus transmission during the two outbreaks in the area [17]. While NDUV, BBKV and Orthobunyaviruses were isolated from the flood water *Aedes*, all the WNV isolates in Garissa and Turkana were obtained from *Cx. univittatus*. This suggests that the cycle of transmission of arboviruses is linked to specific mosquito species associated with flooding of breeding sites which results in hatching of hibernating mosquito eggs in abundance as happens during Rift Valley Fever outbreaks and that West Nile virus transmission is mainly driven by *Cx. univittatus* species in this region. Rift Valley Fever virus was detected in flood water *Aedes* mosquitoes only during the outbreak [17] but none was detected/isolated from mosquitoes collected in this survey (2007–2011). The viruses isolated in this study were also detected in a surveillance performed on mosquitoes collected during the outbreak in 2006/07 [18] further demonstrating that increase in mosquito abundance that drives the Rift Valley Fever epidemics/epizootics in hotspot areas also favors the transmission of other arboviruses even between epidemics. This could be an indication that the infection rates of RVFV during inter-epidemic period may be below detectable levels in mosquitoes.

Only Ndumu virus (from 11 pools) was detected from mosquitoes tested from Marigat with over half of these (6) being recovered from *Culex rubinotus*. This indicates the importance of this marsh breeding mosquito that is found in abundance in estuaries and riverines lakes [19] in the maintenance and transmission of this virus in the region. *Cx. rubinotus* is also known to feed preferably on rodents [19], an indicator that rodents may have a role to play in the circulation of Ndumu virus.

Isolates of Sindbis and the closely related Babanki viruses were recovered from *Culex* species from Naivasha, Kisumu and Budalangi, sites neighboring lakes Naivasha and Victoria. These viruses are usually associated with migratory birds which nest around lakes during their stopover breeding seasons. *Culex* species preferably feed on such birds and may also feed opportunistically on humans. A single isolate of Usutu virus was recovered from a pool of *Culex pipiens* from Kisumu. This is another virus that is associated with birds in its natural cycle. It is known to be widely distributed in Africa and Europe and a recent occurrence of an outbreak among birds in Europe has been reported [20]. These observations are an indication of the potential for these viruses to move via East Africa through the migration of birds with the risk of introduction of new variants in our region.

Further analysis of the Orthobunyavirus isolates revealed that the isolates from *Anopheles funestus* pool sampled in Tana delta and another isolate from Garissa consisted of Ngari virus strains. All the Orthobunyavirus isolates were amplified with the primers targeting sections of the S, M and L segments of Bunyamweravirus and those targeting the M segment of Batai virus. The Ngari virus strains were amplified by all the above primers except the Bunyamwera M segment primers. The results were confirmed by sequencing giving 96% identity with the strain detected in the 1997/98 outbreak in Kenya (9). Bunyamwera virus was also detected in mosquitoes circulating in these same sites. The Bunyamwera viruses were amplified by all the above mentioned primers except the Batai M segment primers. This is an important observation which demonstrates that Ngari virus, first reported in Garissa in northern Kenya during the Rift Valley Fever virus outbreak [9], is co-circulating with Bunyamwera virus in Garissa and other parts of Kenya. Ngari virus has been described as a reassortant virus between Bunyamwera and Batai viruses, having the medium RNA segment from Batai and the small and large segments from bunyamwera viruses. Ngari virus was first detected in Kenya from a hemorrhagic fever case in Garissa and Somalia and subsequently 27% of hemorrhagic fever cases seen during the RVFV outbreak in 1997/98 were due to this virus (9). This constitutes the first report of Ngari virus isolation from mosquito vectors in Kenya. Both Tana-delta and Garissa are home to predominantly pastoralist communities which indicate the possible role of livestock in the ecology of this virus, just like Rift Valley Fever virus. *An. funestus* is an important malaria vector known to be anthropophillic and therefore with a potential to transmit this virus to humans that could lead to NRI virus epidemics. Hence, unlike Rift Valley Fever virus, the risk of transmission of NRI virus to humans through mosquito bite may be greater. In West Africa, Ngari virus has been isolated from diverse mosquito species including *Anopheles* and *Culex*[21]. These findings demonstrate the risk of Ngari virus and other arboviruses causing outbreaks/infections in multiple locations/populations in Kenya, findings that must be brought to the attention of public health authorities to encourage and improve disease detected and prevention. This type of surveillance should also be expanded within the country to identify other sites/ecozones that may be at risk. The detection/isolation of insect only Flaviviruses and the uncharacterized Alphaviruses requires further studies to determine their characteristics in more detail and the role of the flaviviruses in the circulation of the medically important flaviviruses.

## Conclusions

The findings of this 5 year entomologic arbovirus surveillance have demonstrated the active circulation and distribution of a variety of arboviruses of public health importance in parts of Kenya, bringing to the attention of relevant authorities, the potential disease risks attributable to arboviruses in diverse ecosystems and provinces in Kenya. The concentration of the viruses in the pastoral arid to semi-arid eco-zones is an indication that livestock and surrounding wild animals may be important in the amplification of these viruses and the public health authorities need to improve detection of these diseases among the pastoral communities. It is important to note that Ngari virus, recently associated with life threatening hemorrhagic fever is more widely distributed in the country than previously thought. This underscores the need for continued surveillance, mapping of disease risk and creation of awareness concerning the risks among health care providers to improve detection, timely response and prevention. In-depth studies of these viruses including vector competence and molecular and evolutionary studies will shed more light on the dynamics of transmission, molecular epidemiology and the potential for future emergence of serious pathogens.

## Methods

The study was approved by the institutional review boards of the Kenya Medical Research Institute and Walter Reed Army Institute of Research.

### Study sites and site description

**Figure 4:**
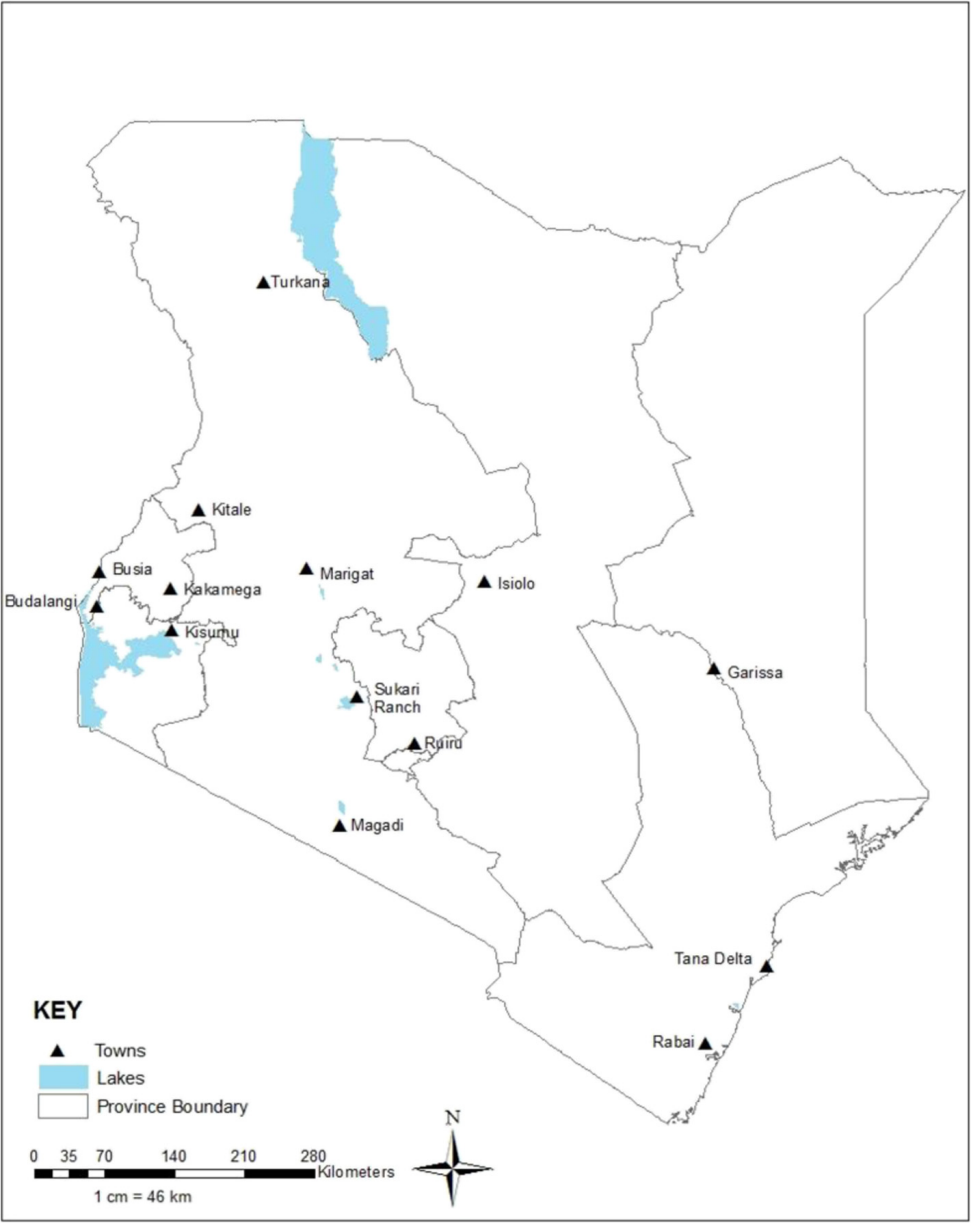
**Map of Kenya.** The map showing the different provinces in Kenya where the mosquitoes that yielded the isolates reported in this manuscript were collected. The study sites were Marigat, Turkana, Magadi and Naivasha (in Rift Valley province); Budalangi, Busia, (in Western province); Kisumu (in Nyanza province); Rabai, Tana-delta and Garissa (in the Coast and North Eastern provinces respectively); Sukari ranch (on the outskirts of Nairobi city in central province).

Mosquitoes were sampled in 11 sites across seven administrative provinces and geographically distinct regions of Kenya (Figure 4) namely Rabai (3°55′0″ N, 39°33′0″ E, elevation: 166 m) and Tana-Delta (02°27′S, 040°17′E, 182 m) in the Coast province. Rabai has an annual mean temperature (AMT) of 26°C, and annual mean rainfall (AMR) of 950 mm and Tana Delta has an AMT of 28°C, AMR of 500 mm. The vegetation comprises of savannah grassland, semi-arid Acacia thorn bushes, and mangrove swamps. Tana-Delta is also prone to occasional flooding when Tana River bursts its banks.

Garissa (0° 27′ 25″ S, 39° 39′ 30″ E, 151 m) is in North Eastern province and falls within arid to semi-arid zone and is predominantly flat with low lying plains. Garissa has an AMT of 28.8°C and AMR of 576 mm and is characterized by infrequent rainfall and are prone to occasional flooding. The vegetation is predominantly *Acacia* type species and *Prosopis juliflora.*

Sukari ranch (1° 15′ 0S, 37° 6′ 0E, 1,585 m) is located on the outskirts of Nairobi city in Central province and is generally cooler, with an AMT of 19°C and AMR of 1,065 mm. In the Rift Valley province, sampling was conducted in Marigat (0° 28′ 0 N, 35° 58′ 60E, 1,062 m), Magadi (1°54′04″ S, 36°17′13″ E, 596 m), and Naivasha (0° 43′ 0S, 36° 25′ 60E, 2,085 m). Marigat, which is located on the shores of Lakes Baringo and Bogoria, has an AMT of 24°C and AMR of 655 mm. Naivasha has an AMT of 25°C and AMR of 750 mm and is located on the shores of Lake Naivasha. Vegetation in Marigat is predominantly *Prosopis juliflora and some acacia*. Magadi, on the shores of Lake Magadi has an AMT of 29°C and AMR of 469 mm. Turkana comprises of both low-lying plains with an altitude range of 370 - 900 m and the mountainous regions ranging from 1,500 – 1,800 m. It lies in the arid and semi arid zone with an AMT temperature of 30°C and an AMR of 300 mm.

Kisumu (1° 18′ 0S, 37° 21′ 0E, 1544 m) lies on the shores of Lake Victoria in Nyanza province and has an AMT of 23°C and AMR of 1,427 mm. It borders Western province where we have the low plains of Busia (0° 28′ 0 N, 34° 6′ 0E, 1,206 m) and Budalangi (0° 08′ 13″ N, 34° 01′ 37″ E, 1,265 m) also on the shores of Lake Victoria. Kakamega (0° 16′ 60 N, 34° 45′ 0E, 1,523 m) and Mt. Elgon (0° 51′ 40″ N, 34° 49′ 07″ E, 2,083 m) are humid with heavy rainfall all year round probably influenced by the Kakamega tropical rainforest. Busia district is the warmest with an AMT of 25°C and AMR of 1,300 mm.

### Site selection

Eight of the sites were selected based on historical arbovirus outbreaks, prior cases or reports of human cases of fevers of unknown origin and an ecological environment suitable for arbovirus transmission (determined by availability of abundant vector habitats). The three remaining sites: Magadi, Turkana and Tana-Delta, were sampled in response to reports of increased flooding and reported upsurge of cases of febrile illnesses. All collection sites were mapped by determining the coordinates (latitude and longitude) on a GPS-12 geographical positioning system (GARMIN International, Kansas, US).

### Mosquito collection

Mosquitoes were sampled only during wet periods when high mosquito population density is expected. Carbon dioxide-baited CDC light traps and landing collection methods were used to collect mosquitoes. Consent to participate in human landing mosquito collection was obtained from willing volunteers. The CDC light traps were hung at least two meters from the ground and baited with carbon dioxide held in igloos next to the traps [22] and left on site from dusk to dawn. Mosquitoes were retrieved from the traps early in the morning and transported to the field laboratory. Volunteers and staff after being trained positioned themselves outside homes and performed landing collections by exposing one leg from which they picked any landing mosquito. Mosquitoes were collected in single vials and transported to the field lab. All sampled mosquitoes were knocked down, packed in labeled 15 ml vials and transported to the laboratory in dry ice or liquid nitrogen. In the laboratory, mosquitoes were sorted, identified and pooled (1 to 25 mosquitoes per pool) by species, sex and collection sites using mosquito identification keys by [23-26] and preserved in 1.5 ml cryogenic vials in -80°C for testing.

### Mosquito processing

Pools of mosquitoes were homogenized in a biosafety level 2 laboratory based at the Kenya Medical Research Institute’s (KEMRI) Centre for Virus Research. One, 4.5-mm-diameter, copper-clad steel bead (BB-caliber airgun shot) was placed in eppendorf tube with the pool of mosquitoes and 1 ml diluent Eagle’s Minimum Essential Media (MEM) (Sigma-Aldrich, St. Louis, MO) with Earle’s salts and NaHCO_3_, supplemented with 15% heat-inactivated fetal bovine serum (FBS), (Sigma-Aldrich), 2% L-Glutamine (Sigma-Aldrich), and 2% antibiotic/antimycotic solution with 10,000 units penicillin, 10 mg streptomycin and 25 μg amphotericin B per ml (Sigma-Aldrich) and shaken vigorously. The supernatant was then harvested by centrifugation in a refrigerated bench-top centrifuge at 12000 rpm for 10 minutes and stored in a 1.5 ml cryovial at -80°C for testing.

### Cell culture inoculation

All mosquito pool supernatants were screened for viruses by cell culture inoculation in Vero cells (monkey kidney epithelial cells). Fifty microliters of each mosquito pool suspension was inoculated into a single well of a 24-well culture plates containing a monolayer of Vero cell cultures in growth medium (Minimum Essential Medium (MEM) with Earles salts, with 10% Fetal Bovine Serum (FBS), 2% Glutamine, 100 units/ml of penicillin, 100 ug/ml streptomycin and 1 μl/ml of Fungizone). Inoculated cultures were incubated at 37°C for one hour to allow virus adsorption. 1 ml of Maintenance medium was then added (MEM with Earles salts with 5% FBS, 2% Glutamine, 100 units/ml of penicillin, 100 ug/ml streptomycin and 1 μl/ml of Fungizone). Cells were incubated at 37°C and observed daily for cytopathic changes. Cell culture supernatants were harvested when cytopathic effect (CPE) involving 50% of the cell monolayer was observed. Due to the costs involved in this screening, only 10% of the negative cultures from the first inoculation were subcultured (including those that were not clearly negative) to improve chances of virus recovery. A virus isolate was suspected when the CPE was reproducible in a passage of the initially harvested culture. The supernatants from all cultures showing reproducible CPE were analyzed further.

### Detection and analysis by RT-PCR

RNA was extracted from a 250 μL of each cell culture supernatant using the Trizol®-LS - Chloroform extraction method [27]. The final RNA pellet was dissolved in 11 μL of nuclease-free water at room temperature and stored on ice or frozen at −80°C ready for RT-PCR. To convert extracted RNA into cDNA, 10 μL of RNA and 2 μL of random hexamer (100 nmol) were combined in a dome-topped PCR tube and placed in a thermal cycler programmed at 70°C for 10 minutes to denature the sample then cooled to 4°C for five minutes. To the tubes, the following components were added: 4 μl of 5x first strand buffer (Invitrogen), 0.01 μmoles of dNTPs (Invitrogen), 0.02 μmoles of DTT (Invitrogen), 10 U of RNase Out inhibitor (Invitrogen) and 100 U of SuperScript III reverse transcriptase (Invitrogen) and incubated in the thermocycler at the following conditions: 25°C for 15 min, 42°C for 50 min, 70°C for 15 min and 4°C hold temperature. The final volume for this reaction was 20 μl which was used for various PCR amplifications using primers targeting virus genera or specific arboviruses. The PCR amplification of targeted viral sequences in the cDNA was performed in a 25-μL reaction containing: 12.5 μl of Amplitaq Gold PCR master mix (Applied Biosystems), 25 picomoles each of forward and reverse primer, 2 μl of the cDNA and 9.5 μl of water to top up to 25 μl. The cDNA was tested using primers targeting flavivirus, alphavirus, and orthobunyavirus arbovirus genera (Table 1). For a sample which tested positive with family or genus primers, it was tested further with primers that target conserved genes in the specific viruses belonging to the genus in question (Table 1). Where a sample was positive by orthobunyavirus, flavivirus or alphavirus primers the genus amplicon was sequenced using both the forward and reverse genus primers.

**Table 1:** DNA sequences of the primers used in this study, their target genes/proteins and positions

	**Virus**	**Gene/protein target**	**Primer sequence**	**Position**	**Reference**
1.	Dengue	Structural polyprotein	D1; (5′-TCA ATA TGC TGA AAC GCG CGA GAA ACC G-3′)	38-65	[28]
	“	“	D2; (5′-TTG CAC CAA CAG TCA ATG TCT TCA GGT TC-3′)	455-483	
2.	Alphavirus	NSP4	VIR 2052 F;(5′-TGG CGC TAT GAT GAA ATC TGG AAT GTT-3′)	6971–6997	[29]
	“	“	VIR 2052R; (5′-TAC GAT GTT GTC GTC GCC GAT GAA-3′)	7086–7109	
3.	Flavivirus	NS5	FU 1; (5′- TAC AAC ATG ATG GGA AAG AGA GAG AA-3′)	9007-9032	[30]
	“	“	CFD2; (5′- GTG TCC CAG CCG GCG GTG TCA TCA GC-3′)	9308-9283	
4.	bunyavirus	nucleocapsid protein	BCS82C; (5′-ATG ACT GAG TTG GAG TTT CAT GAT GTC GC-3′)	86-114	[31]
	“	“	BCS332V; (5′-TGT TCC TGT TGC CAG GAA AAT-3′)	309-329	
5.	Ndumu	envelope (E1) gene	ND 124 F; (5′-CAC CCT AAA AGT GAC GTT-3′)	124-141	[30]
	“	“	ND 632R; (5′- ATT GCA GAT GGG ATA CCG-3′)	615-632	
6.	West Nile	helicase	WN1315F; (5′-GCC AA TTT GCC TGC TCT AC-3′)	1315-1333	[32]
	“	“	WN1824R; (5′-CCA TCT TCA CTC TAC ACT TC-3′)	1824-1843	
7.	Babanki	E1 envelope glycoprotein	Bab 3368 F; (5′- CAG CAG ATT GCG CGA CTG ACC-3′)	3368-3388	[30]
	“	“	Bab 4203R; (5′- GCT CAC GAT ATG GTC AGC AGG-3′)	4184-4203	
8.	Batai	Polyprotein M segment	BATAIM3F; (5′-CCTGGGGAAGCATTGTGATTACT-3′)	852-874	[33]
	“	“	BATAIM3R; (5′-CTAGCCAGCGACTCTTGCCTTCC-3′)	2084-2103	
9.	Sindbis	Non Structural Protein	SINV1; (5′-TTTAGCGGATCGGACAATTC-3′)	5194-5213	[34]
	“	“	SINV2; (5′-GCGGTGACGAACTCAGTAG-3′)	6482-6500	
10.	RVF	Glycoprotein M gene	RVF1;(5′-GAC TAC CAG TCA GCT CAT TAC C-3′)	777-798	[34]
	“	“	RVF2;(5′-TGT GAA CAA TAG GCA TTG G-3′)	1309-1327	
11.	Chikungunya/Onyon’gnyon’g	5′NTR	CHIK3F; (5′-CACACGTAGCCTACCAGTTTC-3′)	14-112	[35]
	“	“	CHIK3R; (5′-GCTGTCAGCGTCTATGTCCAC-3′)	“	
	“	“	ONN3F; (5′-GATACACACACGCAGCTTACG-3′)	11-97	
	“	“	ONN3R; (5′-TACATACACTGAATCCATGATGGG-3′)	“	
12.	Yellow Fever	polyprotein	CAG; (5′- CGA GTT GCT AGG CAA TAA ACA CAT TTG GA-3)	43-71	[36]
	“	“	YF7; (5′- AAT GCT CCC TTT CCC AAA TA- 3′)	1293-1312	

### DNA sequencing and phylogenetic analysis

A Positive control cDNA and a no template negative control were included during the setting up of all PCR reactions. Electrophoresis of the amplified DNA products was done on a 1- 2% Agarose gel in Tris-borate EDTA buffer stained with ethidium bromide. The PCR product bands were visualized by a UV transilluminator and recorded using a gel photo imaging system.

Amplified target DNA bands were either purified directly from the PCR reaction or from the gel using Wizard® SV Gel and PCR Clean-Up System kit (Promega). Sequencing was outsourced and performed using ABI-PRISM 3130 Genetic Analyzer (Applied Biosystems, Foster City, CA). The sequences were compared with available sequences using Basic Local Alignment Search Tool [37] and the GenBank database [38] to confirm the identity of the virus isolate. The sequences were aligned using clustalW and Molecular Evolutionary Genetics Analysis (MEGA) software version 5.05 was used for phylogenetic analysis [39] of the nucleotide sequences of the Alphaviruses, Orthobunyaviruses and Flaviviruses in addition to a few selected reference sequences from the genbank database. Bootstrap resampling to determine confidence values on the groupings within trees was performed with one thousand replicates [40].

## Competing interests

The authors hereby declare that they have no competing interests.

## Authors’ contributions

CO contributed in the detection and identification of the virus isolates, data analysis, drafted manuscript, and final manuscript preparation. JL conducted fieldwork, contributed to field study design and contributed to drafting manuscript, and final manuscript preparation. AM, HK and EC performed virus isolation and contributed to the data analysis and review of the manuscript. SY and JM conducted fieldwork, LM, SK, JHR and JB contributed to field and lab study design and final manuscript preparation. DS and EW, the directors of the Global Emerging Infections surveillance and response System/Department of Emerging Infectious Diseases provided overall project management and guidance and ensured continued funding for the project. RS contributed to overall study design, overall supervision of implementation, data analysis, drafted manuscript and final manuscript preparation. All authors read and approved the final manuscript.

## Supplementary Material

Additional file 1 Table showing the total number of mosquitoes collected for each species, the number of pools tested for each species and the viruses isolated from diverse mosquito species and sites. Click here for file
